# Foot Structure of Girls and Boys in the Final Stage of Early Childhood Taking into Account the Half-Yearly Age Ranges

**DOI:** 10.3390/ijerph20010629

**Published:** 2022-12-30

**Authors:** Ewa Puszczalowska-Lizis, Sabina Lizis

**Affiliations:** Medical College, Institute of Health Sciences, University of Rzeszow, Warzywna 1a, 35-959 Rzeszow, Poland

**Keywords:** body posture, foot structure, health behaviors, children

## Abstract

The aim of this study was to analyze the characteristics of foot structure in girls and boys in the final stage of early childhood, taking into account the half-yearly age ranges. The study was carried out among 800 children aged 3. The research tool was the podoscope CQ-ST. The collected research results were analyzed with the use of Student’s *t*-test or Mann-Whitney U test. Statistically significant sex differences in the length of the right and left foot, the width of the right and left foot and Clarke’s angle of the right and left foot in each of the identified age groups were found. There were statistically significant differences in the length of the right and left foot, the width of the right and left foot, and the Clarke’s angle of the right and left foot were noted separately in girls and boys. Sexual dimorphism in foot length, foot width, and Clarke’s angle occurs in 3-year-old children. In each of the age groups, boys are characterized by longer and wider, and lower arched right and left foot. Characteristic features differentiating children in the 1st and 2nd age group are foot length, foot width and Clarke’s angle. Girls and boys in the 2nd age group have longer and wider, and higher arched right and left feet. This indicates a high dynamic of increases in the value of these features.

## 1. Introduction

The human foot is shaped individually for each person, and numerous and complex functions depend on the correct structure and operation of its individual components. The arch of the foot is formed gradually during ontogenesis, with marked periods of increased and slower pace, in accordance with the laws of biological development [1,2,3,4,5,6,7]. The issue of foot shape has been discussed numerous times. However, these studies are fragmentary, usually based on the analysis of single, selected features, which does not provide complex information about the shape of the entire foot. Some authors analyzed the results obtained in studies on a rather unrepresentative sample or without taking into account the criterion of the homogeneity of the examined material, in groups of people with a large age range, ranging from a few to even a dozen or so years. Many studies concern people in development periods characterized by relative stability [8,9,10,11,12,13,14,15,16,17].

Most of the authors focused on assessing the shape of the foot starting from middle childhood, which, according to Kielar-Turska [18], lasts from 4 to 6 years of age. Therefore, in the literature very few reports are available on the formation of the foot in the final stage of early childhood, which is the beginning of dynamic changes. There are few studies which authors have compared the incidence of feet with lowered arching in children in different age groups, in relation to sex, somatic structure, climatic, ethnic and socio-economic conditions. Vergara-Amador et al. [11] based on the study of children from Bogota and Barranquilla, Colombia, showed the relationship between age and the incidence of feet with normal and reduced arches. In children between 3 and 5 years of age, the percentage of feet with reduced arches was higher compared to those between 6 and 7 years of age. The authors treat this condition as an expression of a natural development process related to the formation of an arched structure of the foot with age. Studies by Riddiford-Harland et al. [19] showed that obese children were characterized by a thicker layer of adipose tissue in the metatarsal area compared to peers with a normal body build, while Evans and Karimi [20] excluded the relationship between body weight and flat feet determined on the basis of Foot Posture Index in children aged 3 up to 15 years. Sacco et al. [21], comparing the features of foot structure in children from Germany and Brazil, found a similar development of the longitudinal arch in both populations. Therefore, they excluded the influence of climatic conditions and ethnicity on the development of the longitudinal foot arch in children aged 3–10 years. 

Awareness of the current state of research prompted the authors to undertake the topic of the study, and the main goal was to fill the gap in the scientific literature in the field of the discussed subject. The starting point was the assumption that developmental changes at the beginning of ontogenesis are so dynamic that analyzing foot structure features in children born in the same year, but without taking into account the half-year intervals, is insufficient. Therefore, the aim of the study was to analyze the characteristics of foot structure in girls and boys in the final stage of early childhood, taking into account the half-yearly age ranges. 

Research questions:

1. Do 3-year-old children, in each of the separated half-year ranges, show sexual dimorphism of foot structure features?
2. Are there any differences in the values of foot structure features between children classified into age groups taking into account the half-year ranges?

## 2. Materials and Methods

### 2.1. Participants

The study comprised of 800 children aged 3 (400 girls and 400 boys) attending the randomly selected preschools in the in the Podkarpackie region in South-Eastern Poland. The selected group was subsequently verified in terms of its compliance with pertinent inclusion and exclusion criteria. The qualifying criteria was calendar age in the range of 3.00–3.99, written consent of parents/legal guardians for participation in research. The study excluded children from preterm delivery and those with deformation of the lower limbs, neurological disorders and diseases and/or injuries of the musculoskeletal system, including lower limbs. The basis for exclusion from the tests was also the refusal or reluctance of the child to cooperate in the implementation of research procedures.

According to the assumption, 3-year-olds included children aged between 3.00 and 3.99 years. The mean of the class was 3.5 years. The calendar age, expressed in a decimal system, was the difference between the date of the examination and the date of birth [22]. Subsequently, it was the basis for the qualification of children in the age categories, taking into account the half-year ranges semi-annual division. 

Two age ranges were distinguished:

− age range I (1st group): children aged 3.00–3.49 years (200 girls and 200 boys);
− age range II (2nd group): children aged 3.50–3.99 years (200 girls and 200 boys).

The characteristics of the study group in terms of age are presented in Table 1.

Table 2 contains the characteristics of the somatic features of the examined children. These data indicate no statistically significant sex differences in body weight and height in children classified into particular age groups. Both in the case of girls and boys, statistically significant differences were noted between children classified into age categories taking into account the half-year ranges. Children in the 2nd age group were characterized by greater body weight (girls: *p* < 0.001; boys *p* < 0.001) and body height (girls: *p* < 0.001; boys *p* < 0.001) compared to children in the 1st age group. BMI values did not significantly differentiate 3-year-old girls and boys.

### 2.2. Design

The main research tool was the CQ-ST podoscope (manufactured by Electronic System, Ltd., Czernica, Poland). After entering the podoscope plate, the child assumed a free standing position, with the upper limbs lowered along the trunk. The lower limbs were hip-width apart and the angle of the feet apart was natural, unforced. The person conducting the research controlled the correctness of placing and loading the feet. In the case of incorrect foot loading or loss of balance, the examination was performed again. 

The calculations included following foot features: [13,23,24].

− foot length–the line “L” connecting the most distal point of the forefoot (on the pad of the longest toe) with the farthest point within the hindfoot [cm];
− foot width–the line “W” connecting the most medially located point on the head of the first metatarsal bone (metatarsale tibiale, mtt) with the point lying most laterally on the head of the fifth metatarsal bone (metatarsale fibulare, mtf) [cm];
− Clarke’s angle (Cl)–longitudinal foot arch–is constructed by drawing a tangent to the medial edge of the foot and the line joining the point of the largest recess of the footprint with the mtt point [°];
− heel angle (γ)–transverse foot arch–is comprised between the tangents to the medial and lateral edge of the foot, which cross over the heel [°];
− hallux valgus angle (α)–the angle between the tangent line to the medial edge of the foot and the tangent to the pad of the hallux toe, derived from the mtt point [°];
− the angle of the varus deformity of the fifth toe (β)–the angle between the tangent line to the lateral edge of the foot and the tangent to the pad of the fifth toe, derived from the mtf point [°].

**Figure 1:**
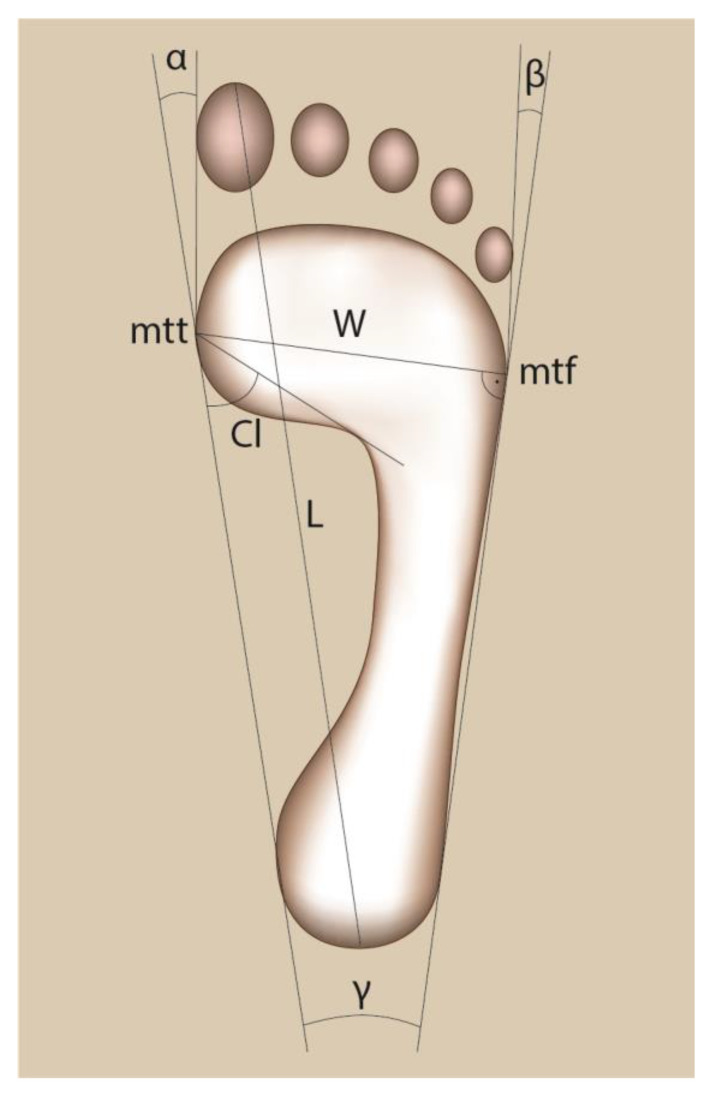
The manner of marking the feet structure indices.

The procedures for calculating the feet features are shown in Figure 1.

Measurements of the body mass (using an OMRON BF500635 medical scale, manufactured by Omron Ltd., Kyoto, Japan), and body height (using a GPM anthropometer, manufactured by Vitako Ltd., Zurich, Switzerland), were taken. The obtained data were used to calculate the Body Mass Index (BMI). 

The examinations were carried out in in accordance with the Helsinki Declaration procedures, in preschool institutions, in a gym and play rooms, in the presence of child educators/guardians. In order to ensure the integrity of the research process, all tests were carried out in the morning, using the same measuring instruments. Children wore their underwear, and were barefooted. The study was conducted according to the guidelines of the Declaration of Helsinki, and approved by the Bioethics Review Committee of the University of Rzeszow, Poland (protocol code 2/2/2017). Parents or legal guardians were advised of the aim, main principles of the study, and on statutory right to opt out of the study protocol at any stage.

### 2.3. Statistical Analysis

The Statistica application, ver. 13.1 PL (StatSoft Inc., Tulsa, OK, USA; StatSoft, Krakow, Poland) was used to process the test results. The Shapiro-Wilk test was used to check if a variable follows a normal distribution. Parametric Student’s *t*-test for independent samples was applied to analyze the data with normal distribution, while the non-parametric Mann Whitney U test was used due to the non-compliance with the normal distribution. The statistical significance was set at *p* < 0.05.

## 3. Results

The data in Table 3 indicate the occurrence of statistically significant gender differences in the length of the right foot (1st group: *p* < 0.001; 2nd group: *p* < 0.001), the length of the left foot (1st group: *p* < 0.001; 2nd group: *p* < 0.001), the width of the right foot (1st group: *p* < 0.001; 2nd group: *p* < 0.001) and the width of the left foot (1st group: *p* < 0.001; 2nd group: *p* < 0.001) in each of the identified age groups. The values of these features in boys were higher than in girls. 

In addition, the comparison of the above-mentioned characteristics within a given sex in children classified into age categories taking into account the half-year age ranges showed statistically significant differences in terms of right foot length (girls group 1st–2nd: *p* < 0.001; boys group 1st–2nd: *p* < 0.001), left foot length (girls group 1st–2nd: *p* < 0.001; boys group 1st–2nd: *p* < 0.001), right foot width (girls group 1st–2nd: *p* < 0.001; boys group 1st–2nd: *p* = 0.005) and left foot width (girls group 1st–2nd: *p* = 0.003; boys group 1st–2nd: *p* < 0.001). Both in the case of girls and boys, the values of these features were higher in those classified into the 2nd age group than in those classified into the 1st age group (Table 3). 

The data in Table 4 indicate the presence of statistically significant sex differences in the Clarke’s angle of the right (1st group: *p* = 0.016; 2nd group: *p* < 0.001) and left (1st group: *p* = 0.020; 2nd group: *p* < 0.001) foot in each of the age groups. The values of this feature in boys were lower than in girls. In addition, in children from the 2nd age group, statistically significant sex differences were noted in terms of the hallux valgus angle of the right (*p* < 0.001) and left (*p* < 0.001) foot. The values of this angle in boys were higher than in girls. 

Statistically significant differences in the values of Clarke’s angle recorded in the 1st and 2nd age groups were found in both girls and boys (right foot: girls group 1st–2nd: *p* < 0.001; boys group 1st–2nd: *p* < 0.001, and left foot: girls group 1st–2nd: *p* < 0.001; boys group 1st–2nd: *p* = 0.002). In both sexes, the values of this index in children from the 2nd age group were higher compared to children from the 1st age group. There was also a statistically significant difference in the values of the hallux valgus angle of the right foot between girls from the 1st and 2nd age group (*p* = 0.036). In girls from the 1st age group, the values of this angle were higher compared to girls from the 2nd age group (Table 4).

## 4. Discussion

In our research there were sex differences in the length and width of the feet, both in the 1st and 2nd age groups. These differences indicate that boys’ feet were longer and wider than girls’ feet. The obtained results are difficult to relate to the reports of other authors. Although the length of the foot is often considered the basis of the unit of measurement that allows you to follow the height of the foot, this issue is addressed piecemeal. The study of Vrdoljak et al. [25] among 2745 preschool children from Zagreb and Split is one of the few. The authors indicated that the dynamics of foot length growth is determined by an exponential curve, however, the differences in the values of this feature in terms of sex are negligible. The lack of studies on inter-sex comparisons of foot width in children in the final stage of early childhood precludes the possibility of discussing the obtained results with the conclusions of other authors. 

We also found sexual dimorphism of the longitudinal arch. In each of the age groups, boys’ feet were characterized by lower values of Clarke’s angle, indicating a lower longitudinal foot arch. This suggests that already at the beginning of the formation of the foot arch, the development of the medial longitudinal arch is slower in them than in girls. One of the few studies on a similar subject is the study by Pffeifer et al. [26] carried out in the United States, where the percentage of flaccid flat feet in the case of 3-year-olds was 54%, while in 6-year-olds the feet with reduced arches were found in 24% of the respondents. Taking into account the gender, the proportions referring to the frequency of this deformity concerned 36% of girls and 52% of boys. On this basis, the authors concluded that boys have a greater tendency to lower the medial edge of the foot than girls. Similarly, Chen et al. [27] showed a relationship between sex and the incidence of flat feet in preschool children. 

In this study, there was also differentiation in terms of foot length and width separately in girls and boys classified into half-yearly age ranges. In both sexes, the feet in the 2nd age group were significantly longer and wider than in the 3-year-olds from the 1st age group. This indicates a high dynamic of increases in the values of the listed features. A similar topic was taken up by Vrdoljak et al. [25], who concluded that the period between 2 and 3 years of age is characterized by the greatest variability of foot length. The authors concluded that from the age of 3 to 6, the foot grows quite evenly, growing in length by about 1 cm per year. In the literature, we do not find reports on the dynamics of foot width development, therefore a discussion at this point is impossible. 

Sexual dimorphism was not evident in the transverse foot arch determined on the basis of the heel angle. There were also no differences between children from the 1st and 2nd age range. Therefore, it can be concluded that this is not a feature differentiating the feet shape of girls and boys in the analyzed period of ontogenesis. Due to the lack of reports on similar topics, it is impossible to compare the obtained data with the results of other studies. 

Taking up the issue of toe placement, it should be mentioned that in the literature a lot of attention is devoted to issues related to the hallux, which plays a large role in transferring body weight during locomotion. Most authors assume that abnormalities in its setting have serious clinical consequences and therefore require early, thorough diagnosis. Lincoln i Suen [28] recognized that some changes in the position of toes in young children are usually benign and resolve spontaneously, and understanding developmental changes is necessary to identify structural abnormalities that require intervention. According to the authors, lateral deviation of the hallux may occur in children at the beginning of locomotion, especially in the face of imbalance of the adductor and abductor muscles of the big toe, which during gait should ensure the correct positioning of the first toe and the entire forefoot. With age, as the strength of the foot muscles increases and motor coordination improves, the toe acquires the correct position. Klein et al. [23] based on the study of children aged 3.00–6.50, attending kindergartens in Austria, noted 14% of cases where the toe angle was above 10°. A significant percentage of children wore shoes that were too short, which, according to the authors, contributed to the formation of hallux valgus. Di Giovanni and Greisberg [29] emphasized the importance of the metatarsal heads in carrying the weight of the body. According to the authors, in the standing position, the head of the first metatarsal bone is involved in transferring about 40% of the body weight, and the rest is distributed to the other metatarsal bones. For the proper range of motion, it is necessary to straighten the fingers and to have proper flexibility and tension of joint capsules, ligaments and muscles. During each step, the hallux bends dorsally, and in the case of its incorrect positioning or lack of sufficient flexibility of soft tissues, overloads and disorders within the metacarpophalangeal joint occur. Wheelwright i wsp. [30] noted that at the beginning of locomotion, the child does not load the feet by rolling them from the heels to the toes, but sets them flat, directing the weight of the body directly to the metatarsus. Therefore, it seems that the first radius of the foot together with the hallux are somewhat limited in the load transfer function. It is only between the ages of 2 and 3 that the gait pattern changes and the child gradually, instead of resting the entire foot on the ground, starts contact with the ground from the heel, and at the age of 6 the gait pattern becomes similar to that of an adult. Taking into account the results of our own research, it can be concluded that in the case of children from the 2nd age group, the sex-differentiating feature is the angle of the hallux valgus. The male sex is characterized by a more valgus setting of the hallux of the left foot. However, despite the above-mentioned changes in the foot load pattern, typical for the early stages of ontogenesis, the position of the hallux is not seriously disturbed. The values of the hallux valgus angle do not differ significantly from the lower limit of the norm, which, according to Lizis [31], ranges from 0–9°. However, the obtained results make us think about the causes of the varus position of the fifth toe in the examined children. The values of this angle in both sexes oscillate around the upper limit of normal. It seems that this condition may be of a temporary nature, which should be considered as a consequence of the functional insufficiency of the muscles responsible for its correct positioning, as well as the physiological, varus position of the foot at the beginning of its loading and the associated increased pressure on its lateral surface. However, reference to earlier own research [32] in a group of 5-year-olds suggests that abnormalities in the positioning of the fifth finger may persist longer. The values of the fifth toe varus angle oscillated around the upper limit of the norm, while in the case of the toe varus angle, as in this study, they did not differ significantly from its lower limit. In addition, Knapik and Mazur [33] observed in pre-school children a clear tendency to deepen the varus of the fifth toe, which was deformed earlier than the hallux. The authors assumed that these changes most often arise as a result of incorrect reactions of the foot with footwear. Own research and reports of the cited authors suggest that the fifth toe, due to its delicate structure, is susceptible to deviation from the correct position and therefore, similarly to the hallux, requires early diagnosis. Attention needs to be paid to the shape of the footwear, including its front and side surfaces. Properly designed footwear should provide foot comfort both in static conditions and while walking. Equally important is learning and practicing the correct loading of the feet both in a standing position and during locomotion. 

Summing up our own research and the reports of other authors, it is worth emphasizing the need to care for the proper development of the feet and constant monitoring of their condition, especially in the case of children in the period of developmental plasticity. Early diagnosis of abnormalities makes it possible to undertake immediate therapeutic intervention. In the light of these observations, the issues raised in the work should be considered valuable. An in-depth analysis of an important problem, which is the formation of feet at the stage of early childhood, was made. The authors are convinced that the obtained results and their interpretation will be used in the diagnosis and prevention of foot deformities. Monitoring the feet condition will allow you to determine the correctness of preventive actions, which should be aimed at creating appropriate conditions for shaping children’s feet from an early age and developing proper movement habits. 

## 5. Conclusions

1. Sexual dimorphism in foot length, foot width, and Clarke’s angle occurs in 3-year-old children. In each of the age groups, boys are characterized by longer and wider, and lower arched right and left foot. Another feature differentiating the feet of girls and boys in the 2nd age group is the hallux valgus angle. The male sex is characterized by a more valgus setting of the hallux of the left foot. 

2. Characteristic features differentiating children in the 1st and 2nd age group are foot length, foot width and Clarke’s angle. Girls and boys in the 2nd age group have longer and wider, and higher arched right and left feet. This indicates a high dynamic of increases in the value of these features. The distinguishing feature of girls in the 1st and 2nd age group is the hallux valgus angle. Girls in the 1st age band are characterized by a more valgus position of the hallux than girls in the 2nd age group.

## Figures and Tables

**Table 1:** The age of the study children, taking into account the half-year ranges.

Group	Girls			Boys			Z	*p*
	$\bar{X}\;\pm \;\mathrm{SD}$	Max−Min	Me	$\bar{X}\;\pm \;\mathrm{SD}$	Max−Min	Me		
1st group	3.25 ± 0.17	3.49–2.19	3.24	3.25 ± 0.14	3.49–3.00	3.24	−0.03	0.973
2nd group	3.75 ± 0.15	4.42–3.50	3.75	3.73 ± 0.15	4.12–3.30	3.74	−1.51	0.132

Note: $\bar{X}$—arithmetical mean value; SD—standard deviation; max—maximum value; min—minimum value; Me—median; Z—value of the Mann-Whitney U test statistic; *p*—probability value.

**Table 2:** The somatic features of the examined children.

Group	Girls			Boys			t/Z	*p*
	$\bar{X}\;\pm \;\mathrm{SD}$	Max−Min	Me	$\bar{X}\;\pm \;\mathrm{SD}$	Max−Min	Me		
**Body mass [kg]**								
1st group	15.58 ± 2.13	24.50–11.00	15.00	16.26 ± 2.37	25.00–11.50	16.00	Z = −0.03	0.973
2nd group	16.73 ± 2.25	22.00–12.00	16.25	17.07 ± 2.13	24.50–12.50	17.00	Z = −1.60	0.110
Girls_1st group_–Girls_2nd group_ Z = −17.30; *p* < 0.001 *				Boys_1st group_–Boys_2nd group_ Z = −3.91; *p* < 0.001 *				
**Body height [cm]**								
1st group	96.71 ± 4.15	109.00–85.50	96.50	98.21 ± 4.28	107.50–83.50	98.50	t = −3.55	0.696
2nd group	100.87 ± 4.05	114.50–91.50	100.50	101.09 ± 3.97	113.00–86.50	101.00	Z = −0.70	0.484
Girls_1st group_–Girls_2nd group_ Z = −5.22; *p* < 0.001 *				Boys_1st group_–Boys_2nd group_ Z = −6.55; *p* < 0.001 *				
**BMI**								
1st group	16.59 ± 1.73	22.60–12.06	16.45	16.82 ± 1.88	29.54–13.16	16.65	Z = −0.99	0.324
2nd group	16.39 ± 1.55	22.68–12.50	16.32	16.68 ± 1.61	21.11–12.98	16.57	Z = −1.68	0.092
Girls_1st group_–Girls_2nd group_ Z = −1.18; *p* = 0.238				Boys_1st group_–Boys_2nd group_ Z = −0.42; *p* = 0.672				

Notes: $\bar{X}$—arithmetical mean value; SD—standard deviation; max—maximum value; min—minimum value; Me—median; t—value of the Student’s *t*-test statistics; Z—value of the Mann-Whitney U test statistic; *p*—probability value. * *p* < 0.001.

**Table 3:** The comparison of the length and width of the feet of 3-year-old girls and boys, taking into account half-yearly age ranges.

Group	Girls			Boys			Z	*p*
	$\bar{X}\;\pm \;\mathrm{SD}$	Max−Min	Me	$\bar{X}\;\pm \;\mathrm{SD}$	Max−Min	Me		
**Foot length of the right foot [cm]**								
1st group	14.67 ± 0.77	17.20–12.90	14.60	15.04 ± 0.77	16.90–13.20	15.00	−4.95	<0.001 *
2nd group	15.12 ± 0.76	17.00–13.40	15.10	15.43 ± 0.78	17.70–13.30	15.40	−3.97	<0.001 *
Girls_1st group_–Girls_2nd group_ Z = −5.80; *p* < 0.001 *				Boys_1st group_–Boys_2nd group_ Z = −4.68; *p* < 0.001 *				
**Foot length of the left foot [cm]**								
1st group	14.69 ± 0.77	17.20–12.90	14.65	15.05 ± 0.77	17.00–13.20	15.00	−4.95	<0.001 *
2nd group	15.13 ± 0.76	17.10–13.50	15.10	15.45 ± 0.78	17.70–13.10	15.40	−4.13	<0.001 *
Girls_1st group_–Girls_2nd group_ Z = −5.52; *p* < 0.001 *				Boys_1st group_–Boys_2nd group_ Z = −4.71; *p* < 0.001 *				
**Foot width of the right foot [cm]**								
1st group	5.60 ± 0.35	7.00–4.80	5.60	5.82 ± 0.36	7.10–4.90	5.80	−6.15	<0.001 *
2nd group	5.73 ± 0.31	6.40–4.70	5.80	5.92 ± 0.34	6.80–5.00	5.90	−5.43	<0.001 *
Girls_1st group_–Girls_2nd group_ Z = −4.34; *p* < 0.001 *				Boys_1st group_–Boys_2nd group_ Z = −2.81; *p* = 0.005 *				
**Foot width of the left foot [cm]**								
1st group	5.67 ± 0.38	7.00–4.80	5.70	5.91 ± 0.38	7.00–5.00	5.90	−6.11	<0.001 *
2nd group	5.78 ± 0.35	6.70–5.00	5.80	5.98 ± 0.36	7.10–5.00	6.00	−5.36	<0.001 *
Girls_1st group_–Girls_2nd group_ Z = −3.01; *p* = 0.003 *				Boys_1st group_–Boys_2nd group_ Z = −1.99; *p* < 0.001 *				

Notes: $\bar{X}$—arithmetical mean value; SD—standard deviation; max—maximum value; min—minimum value; Me—median; Z—value of the Mann-Whitney U test statistic; *p*—probability value. * *p* < 0.001.

**Table 4:** The comparison of characteristics determining the longitudinal and transverse foot arches as well as the position of the hallux and Vth toe in 3-year-old girls and boys, taking into account half-yearly age ranges.

Group	Girls			Boys			Z	*p*
	$\bar{X}\;\pm \;\mathrm{SD}$	Max−Min	Me	$\bar{X}\;\pm \;\mathrm{SD}$	Max−Min	Me		
**Clarke’s angle of the right foot [°]**								
1st group	21.93 ± 10.10	47.00–0.00	22.50	19.25 ± 9.90	40.00–0.00	19.00	−2.40	0.016 *
2nd group	29.96 ± 10.77	47.00–0.00	33.00	23.60 ± 10.69	48.00–0.00	23.00	−5.91	<0.001 *
Girls_1st group_–Girls_2nd group_ Z = −7.49; *p* < 0.001 *				Boys_1st group_–Boys_2nd group_ Z = −3.77; *p* < 0.001 *				
**Clarke’s angle of the left foot [°]**								
1st group	20.69 ± 9.94	47.00–0.00	20.00	18.17 ± 10.30	40.00–0.00	18.50	−2.34	0.020 *
2nd group	29.20 ± 11.39	47.00–0.00	32.00	21.91 ± 11.46	46.00–0.00	21.00	−6.17	<0.001 *
Girls_1st group_–Girls_2nd group_ Z = −7.58; *p* < 0.001 *				Boys_1st group_–Boys_2nd group_ Z = −3.16; *p* = 0.002 *				
**Heel angle (γ) of the right foot [°]**								
1st group	16.43 ± 2.16	24.00–11.00	16.00	16.60 ± 1.99	22.00–12.00	16.00	−1.08	0.281
2nd group	16.33 ± 1.79	21.00–12.00	16.00	16.49 ± 1.95	21.00–11.00	16.00	−0.43	0.669
Girls_1st group_–Girls_2nd group_ Z = −0.01; *p* = 0.997				Boys_1st group_–Boys_2nd group_ Z = −0.69; *p* = 0.487				
**Heel angle (γ) of the left foot [°]**								
1st group	16.37 ± 1.20	21.00–10.00	16.00	16.62 ± 2.15	23.00–12.00	16.50	−1.08	0.281
2nd group	16.15 ± 1.96	20.00–10.00	16.00	16.34 ± 2.18	22.00–12.00	16.00	−0.60	0.549
Girls_1st group_–Girls_2nd group_ Z = −0.77; *p* = 0.439				Boys_1st group_–Boys_2nd group_ Z = −1.16; *p* = 0.247				
**Hallux valgus angle (α) of the right foot [°]**								
1st group	2.99 ± 3.30	12.00–0.00	2.00	3.74 ± 3.78	16.00–0.00	3.00	−1.89	0.059
2nd group	2.32 ± 3.01	13.00–0.00	0.00	3.82 ± 3.63	14.00–0.00	3.00	−4.36	<0.001 *
Girls_1st group_–Girls_2nd group_ Z = −2.09; *p* = 0.036 *				Boys_1st group_–Boys_2nd group_ Z = −3.69; *p* = 0.713				
**Hallux valgus angle (α) of the left foot [°]**								
1st group	3.59 ± 3.94	14.00–0.00	2.50	4.17 ± 4.16	19.00–0.00	3.50	−1.55	0.121
2nd group	2.96 ± 3.80	16.00–0.00	0.00	4.47 ± 4.07	16.00–0.00	4.00	−4.08	<0.001 *
Girls_1st group_–Girls_2nd group_ Z = −1.74; *p* = 0.082				Boys_1st group_–Boys_2nd group_ Z = −0.87; *p* = 0.384				
**The angle of the varus deformity of the fifth toe (β) of the right foot [°]**								
1st group	8.62 ± 5.88	26.00–0.00	8.00	8.56 ± 5.13	25.00–0.00	9.00	−0.28	0.780
2nd group	9.25 ± 4.78	23.00–0.00	9.00	8.95 ± 5.19	22.00–0.00	9.00	−0.53	0.594
Girls_1st group_–Girls_2nd group_ Z = −1.68; *p* = 0.094				Boys_1st group_–Boys_2nd group_ Z = −0.81; *p* = 0.419				
**The angle of the varus deformity of the fifth toe (β) of the left foot [°]**								
1st group	8.82 ± 5.15	26.00–0.00	9.00	8.69 ± 4.79	22.00–0.00	9.00	−0.27	0.788
2nd group	8.50 ± 4.93	21.00–0.00	9.00	8.20 ± 5.15	19.00–0.00	8.00	−0.62	0.538
Girls_1st group_–Girls_2nd group_ Z = −0.42; *p* = 0.674				Boys_1st group_–Boys_2nd group_ Z = −0.83; *p* = 0.407				

Notes: $\bar{X}$—arithmetical mean value; SD—standard deviation; max—maximum value; min—minimum value; Me—median; Z—value of the Mann-Whitney U test statistic; *p*—probability value. * *p* < 0.001.

## Data Availability

Even though the source datasets analyzed in this article are not publicly available, they may be made available to the researchers by the Corresponding Author upon reasonable request, subject to the applicable legal restrictions in place.
